# The Effects of Respiratory Vaccine Type and Timing on Antibody Titers, Immunoglobulins, and Growth Performance in Pre- and Post-Weaned Beef Calves

**DOI:** 10.3390/vetsci10010037

**Published:** 2023-01-04

**Authors:** Jeff M. Matty, Cassidy Reddout, Jordan Adams, Mike Major, David Lalman, Rosslyn Biggs, Janeen L. Salak-Johnson, Paul A. Beck

**Affiliations:** 1 Department of Animal and Food Sciences, Oklahoma State University, Stillwater, OK 74078, USA; 2 Department of Animal Science, Texas A&M University, College Station, TX 77843, USA; 3 Veterinary Clinical Sciences Department, College of Veterinary Medicine, Oklahoma State University, Stillwater, OK 74078, USA

**Keywords:** bovine respiratory disease, beef calves, weaning, preconditioning, vaccination

## Abstract

**Simple Summary:**

Different bovine respiratory vaccine antigen types and timing schedules were utilized to determine the impact on antibody titers, immunoglobulins, and performance in beef cattle. These results suggest that vaccinating calves with a killed viral (KV) vaccine at 2 to 3 months of age and not re-vaccinating per manufacturer specifications did not provide an immune titer response and thus may not provide the preweaning disease protection desired. Vaccination with a modified live (MLV) vaccine appeared to produce a more robust antibody response, even in the face of maternal interference and when given as a booster to animals who previously received a KV vaccination. Delay of initial vaccination (MLV) until weaning may have delayed antibody production and resulted in a lower Th-2 (antibody-mediated) immune response.

**Abstract:**

In order to examine the effects of vaccine type and timing of crossbred beef calves (*n* = 151) were assigned to one of three BRD vaccination protocols stratified by breed of sire, sex, and date of birth, which included: (1) KM—a pentavalent killed viral (KV) vaccine at 2 to 3 months of age (D 0) and a pentavalent modified-live viral (MLV) vaccine at weaning (D 127); (2) MM—MLV on D 0 and revaccinated on D 127 or (3) WN—MLV at weaning and D 140. Vaccination treatment did not affect performance nor BRSV serum-neutralizing antibody titers. Serum-neutralizing antibody titers to BVDV-1 were greatest for the MM through D 154. However, following booster (KM) or initial vaccination (WN) at D 127, titers increased for the other treatment groups to higher values (KM) by the end of the study. Delay of initial vaccination until weaning may have delayed specific antibody response in the WN group and skewed the immune response towards a Th-1 or cell-mediated response. Overall, the inclusion of an MLV in the vaccine protocol resulted in a more robust antibody response, and the timing of vaccination may affect the onset of efficacious and robust vaccine responses.

## 1. Introduction

Preconditioning practices among U.S. beef cattle were first conceived in the mid-1960s to minimize adverse effects associated with common stressors such as weaning and commingling of calves upon entry into the feedlot [1]. During the early years, preconditioning protocols were highly varied. More recently, most preconditioning programs have adopted a standardized set of protocols with minimum requirements of a 45-day weaning period, allowed vaccination protocols, castration, dehorning, and acclimation to a concentrated supplemented diet [2]. The preconditioning period allows the calf time to overcome the physiological stress associated with weaning and transportation [2]. Weaning stress can negatively impact feed intake and weight gain. It may also disrupt vaccine efficacy and subsequent titer production [3], implying that the timing of vaccination may be a vital component of the success of immunization during vulnerable periods. Prolonged exposure to stress can suppress the immune system, opening the possibility of pathogenic infection. Stress can prevent animals from fully expressing immunity when vaccinated [3], so many vaccination protocols are designed to administer the initial vaccination preweaning at a time of lower stress than if the administration is at weaning [4]. A vaccination protocol is a potential preventative measure to control the spread of bovine respiratory disease in beef calves.

Bovine respiratory disease (BRD) vaccines vary in antigen and antigen type. Killed viral (KV) and modified-live viral (MLV) vaccines stimulate the immune system via different mechanisms. An MLV vaccine stimulates T cells to drive a cell-mediated immune response and B cells to produce an antibody response to protect from pathogen exposure [5]. At the same time, a KV vaccine stimulates B cells by activating a humoral immune response, producing antibodies against specific antigens in the vaccine [6]. Killed viral vaccines use an inactivated form of the virus to initiate an immune response and are often combined with an adjuvant to increase the stimulation of immune function. According to the manufacturer label, most KV vaccines require a booster dose to achieve their full potential. One common issue amongst calves receiving the primary dose of the KV vaccine at branding is the failure to receive the booster dose in the time specified on the manufacturer’s instructions [2]. In contrast, MLV can stimulate the immune system after a single dose. Killed viral vaccines elicit a humoral immune response that is primarily antibody specific. Given the mechanism by which KV elicits an immune response, KV administered in the presence of high maternal antibodies may form an antibody/antigen complex, neutralizing the vaccine antigen and preventing an antibody response [7]. Modified-live vaccines generate both a humoral and a cell-mediated immune response. Like KV, maternal antibodies may also neutralize the humoral response to MLV. However, even when high levels of circulating maternal antibodies are present, a cell-mediated immune response to MLV antigen can effectively establish immunization in the young animal [7].

Timing strategies for administering vaccines should be optimized as maternal antibodies, poor nutrition, and stress may decrease vaccine efficacy [8,9,10,11]. In young calves, dependency on passive acquired colostral antibodies is vital to survival; however, colostral antibodies can interfere with the calf’s ability to develop and mount an immune response to vaccine antigens [8]. Differences in vaccination timing may relate to producer convenience, preference, and an attempt to protect the reproductive efficiency of the dam. Vaccination failures are often due to management constraints [9,10], and research is needed to determine the most effective vaccination type and timing combinations.

Despite decades of research, limited progress has been made toward controlling the spread of BRD. Research indicates vaccine efficacy and efficiency, but BRD remains the costliest disease in feedlot cattle and breeding stock. Perhaps the answer to disease prevention is the timing of vaccine administration rather than the vaccine itself. Numerous studies examine the timing of vaccine administration in neonates, preconditioned and receiving cattle [4,8,9,10,11,12]. Here, our study followed vaccination protocols that align with the Oklahoma Quality Beef Network, the VAC45 preconditioning program [2], and vaccination strategies commonly used by cow-calf producers. Our goal was to determine antibody response to BRD vaccination while focusing on vaccine type and timing under field conditions. Therefore, the objective of our study was to examine the effects of vaccination timing and vaccine type on BVDV and BRSV serum-neutralizing antibody titers and body weight performance in pre-and post-weaned beef calves.

## 2. Materials and Methods

All animal work was conducted in strict accordance with Oklahoma State University’s Institutional Animal Care and Use Committee (Protocol #20-34).

### 2.1. Treatment and Vaccination Procedures

One hundred and fifty-one Angus, Angus x Hereford, or Charolais x Angus calves (*n* = 67 heifers and *n* = 84 steers) were used to examine the effects of vaccine type and timing on animal performance, morbidity and antibody response pre- and postweaning. Calves were assigned to one of three vaccination protocols stratified by breed of sire, sex, and date of birth. Vaccination treatments included: (1) a pentavalent-killed viral (KV; ViraShield 6, Elanco US Inc., Greenfield, IN, USA) vaccine at 2 to 3 months of age (D 0) and revaccinated with a pentavalent modified-live viral (MLV; Titanium 5, Elanco Animal Health, Greenfield, IN, USA) vaccine at weaning (D 127) (KM; *n* = 52); (2) MLV vaccine on D 0 and revaccinated on D 127 (MM; *n* = 49) or (3) MLV on D 127 and revaccinated on D 140 (WN; *n* = 46). Vaccines were administered subcutaneously using Beef Quality Assurance guidelines [13] at the recommended dose of 5 mL for KV and 2 mL for MLV. Vaccines are labeled as preventative against diseases caused by infectious bovine rhinotracheitis, bovine viral diarrhea type 1 and type 2, parainfluenza type 3, and bovine respiratory syncytial virus.

### 2.2. Animal Management

Calves used in this experiment were born between 12 February and 27 April 2020, at Oklahoma State University’s Range Cow Research Center—South Range Unit near Stillwater, Oklahoma (Latitude 36.1226, Longitude −97.2492, Elevation 965 ft.). At the start of the study (D 0), the calves were 69.0 ± 37.5 days of age, and body weight (BW) was 110.5 ± 7.52 SD, kg and 107.8 ± 7.15 SD, kg for steers and heifer calves, respectively. The cow herd was grouped based on parity. The first group consisted of first- and second-parity heifers and multiparous mature cows (>6 years) at the range headquarters (HQ, *n* = 89), and the second group consisted of three- to five-year-old multiparous cows (SEC, *n* = 62). The groups were housed on separate ranches approximately 3.2 km apart. Dams were vaccinated against BRD 30 to 45 d prior to spring breeding in the year prior to the birth of trial calves with the MLV vaccine; however, dams were not revaccinated during our study. The stocking rate at HQ was 1 cow-calf pair per 3.25 ha, and at SEC stocking rate was 1 cow-calf pair per 4 ha. The forage resources included a variety of warm-season grasses, including bermudagrass (*Cynodon dactylon*), dallisgrass (*Paspalum dilatatum*), crabgrass (*Digitaria sanguinalis*), and tallgrass prairie native species (primarily big—bluestem (*Andropogon gerardii*); little bluestem (*Schizachyrium scoparium*); indiangrass (*Sorghastrum nutans*)).

On D 0, calves were separated from their dam, weighed, blood collected via jugular venipuncture, vaccine administered (if appropriate), and then returned to their dams until weaning (D 127). At each ranch on D 0, all calves were comingled and brought through the working facility in random order with no regard for treatment. All treatments were represented on both ranches of origin, and steps were taken to ensure no transfer of any residual vaccine across the three regimens by using separate needles and syringes for each vaccination treatment. At weaning, all calves were weighed, blood collected, and vaccine treatment applied, and then calves were kept for one week in a 0.61 ha pasture at their birth location for fence-line weaning [14]. Fence-line weaning management was conducted as described by Price et al. [14] to reduce the stress from dam separation. Briefly, cows and calves were rotated into the weaning pasture, the next day, cows and calves were separated, and calves returned to the weaning pasture with cows placed in the adjacent pasture separated by a steel pipe fence. After the one week of fence-line weaning, SEC calves were transported to the HQ weaning facilities and commingled in a 0.61-ha pen for one week, then moved to a 15-ha paddock which consisted mostly dormant mixed grass (native and bermudagrass). Fourteen days postweaning (d 140), calves in the WN treatment were revaccinated with the same MLV vaccine type previously used. The weaned calves were fed a supplemental concentrate containing monensin (Rumensin, Elanco US Inc.) at an average of 0.5% of BW and top dressed with a corn-based coccidiostat (Deccox, Zoetis Animal Health) for the first 30 days from the onset of weaning and provided ad libitum access to mixed grass hay and fresh water. Subsequent calf BW and blood collection measurements occurred on days 140, 154, 168, and 182. 

Upon arrival at the HQ facility (D 134), all calves were treated for external parasites using a pour-on dewormer (Cydectin, Elanco Animal Health, Greenfield, IN, USA) and internal parasites with an oral drench (Safeguard, Merck Animal Health), vaccinated with a multivalent clostridial bacterin-toxoid (Vision 7, Merck Animal Health). A coccidiosis preventative (Corid, Huvepharma Inc., Peachtree City, GA, USA) was added to the drinking water for the first five days after arrival at HQ. On D 140, all calves were vaccinated against *Mannheimia haemolytica* (Nuplura PH, Elanco Animal Health, Greenfield, IN, USA). Calves were observed each morning (0730 h) by experienced university personnel for clinical signs of respiratory illness. Evaluators were blinded to treatments, and cattle were not visually identifiable based on treatment during observation. Calves were evaluated daily and scored using the DART system [15] if clinical symptoms were present. Calves receiving a score ≥3 were pulled, and rectal temperatures were measured. Animals displaying rectal temperature ≥40 °C were administered antibiotic treatment.

### 2.3. Blood Collection and Serology

One blood sample was collected from each calf at six-time points on days 0, 127, 140, 154, 168, and 182 via jugular venipuncture into a 10 mL evacuated tube without additive (Monoject, Covidien, Mansfield, MA, USA). Blood samples were stored in an insulated cooler with ice packs. Samples were allowed to clot at room temperature and then centrifuged at 2100× *g* for 20 min at 4 °C. Post centrifugation, serum was extracted and transferred to 2 mL microtubes and stored at −20 °C until serological analysis.

Serology was performed at the Oklahoma Animal Disease Diagnostic Laboratory (Stillwater, Oklahoma). A modified microtiter virus neutralization test measured antibody titers against bovine viral diarrhea virus type 1 (BVDV-1) and BRSV [14]. Briefly, serum samples were diluted 2-fold using Dulbecco’s minimum essential medium (DMEM) in 96-well microtiter plates. An equal volume (25 µL) of virus diluted in DMEM to contain about 100 TCID50/25 µL was added to all sample wells. After incubating the serum/virus mixtures for 1 h, a cell suspension of MDBK cells containing about 10^4^ cells in DMEM containing 10% fetal bovine serum was added to each well. Plates were incubated for 3 days at 37 °C, and wells were examined for virus-specific cytopathic effects (CPE). Titers were expressed as the reciprocal of the highest dilution of serum that completely neutralized the virus.

### 2.4. Serum Immunoglobulins

Immunoglobulin-G (IgG) subsets IgG1 and IgG2 concentrations were measured using a commercially available bovine IgG1 (E11-16) and IgG2 (E11-17) ELISA kits (Bethyl Laboratories Inc., Montgomery, TX, USA), following the manufacturer’s protocol. Briefly, samples were diluted at 1:200,000. Samples and standards were added in duplicate onto 96-well microtiter plates coated in sample diluting buffer with either anti-bovine IgG1 or IgG2 antibody. Plates were incubated at room temperature for 1 h and then washed four times. Anti-IgG1 or anti-IgG2 detection antibody was pipetted into the wells. Plates were incubated at room temperature for one hour, washed four times, and added horseradish peroxidase solution to each well. Plates were incubated at room temperature for 30 min and washed. TMB substrate was added, and plates were incubated in the dark for 30 min at room temperature. The reaction was stopped using a provided solution, and plates were read at 450 nm using a microplate reader (BioTek Epoch, Winooski, VT, USA). A standard curve was used to determine the concentration of the unknown samples using the Gen5 Data Analysis Software (BioTek). Total IgG was calculated by adding the values of the concentration of IgG1 and IgG2. The minimal detectable concentration of both assays was 1.0 ng/mL.

### 2.5. Statistical Analysis

Experimental data were analyzed as a randomized complete block design using PROC MIXED of SAS (SAS Institute, Cary, NC, USA). The calf was identified as the experimental unit and the sampling unit. Blocks were the pastures (HQ and SEC). Calves were designated to a block based on their birth pasture. Calves from HQ were assigned to block one, and SEC calves were assigned to block two. Birthdate, sex, and age of the dam were used as covariates in the BW and average daily gain (ADG) analysis. Treatment, birthdate, age of dam, sex, and treatment × sex interaction were included in the model statement. The random block was used in the random statement. Blood constituent data were analyzed as repeated measures. Treatment, day, age of dam, treatment × day, treatment × sex, and treatment × sex × day interactions were included in the model statement. Significance was observed at (*p* < 0.05). Virus-specific antibody titers were tested for normality of distribution using PROC UNIVARIATE of SAS, and nonparametric data were log_2_ transformed and statistically analyzed as a repeated measure with day and treatment and their interaction as fixed effects and sampling date in the repeat statement.

## 3. Results

During the study, no mortality was reported. Seventeen calves were treated with antibiotics for infectious pododermatitis. Five calves (*n* = 4, KM and *n* = 1 MM groups) were treated once with an antibiotic for respiratory illness determined by dullness, inappetence, nasal discharge, and rectal temperature ≥40 °C. Finally, SN data from four calves were from the study; 2 were removed due to missing data from inadequate serum volume and 2 due to unreadable samples.

### 3.1. Body Weight and Average Daily Gain

The effects of vaccine type and timing treatments on BW and ADG are presented in Table 1. There was no sex × vaccination treatment interaction (*p* ≥ 0.13) for BW or ADG throughout the experiment; thus, only the main effects of vaccination type and timing are presented for these measures. As is commonly expected, BW of heifers was less (*p* ≤ 0.03) than steers preweaning, at weaning on D 127, and throughout the postweaning period. There was no overall effect of vaccination treatment on BW at any point during the experiment (*p* ≥ 0.70).

Preweaning ADG from D 0 to D 127 was less for heifers than steers, but ADG preweaning was not affected by vaccination timing or vaccine type. Likewise, ADG was not affected (*p* = 0.63) by vaccination treatment after weaning from D 127 to D 140, following the initial MLV vaccination of the calves in WN treatment and booster revaccination of calves in KM and MM treatments. There was a tendency (*p* = 0.08) for reduced ADG from D 140 to D 154 by calves in WN treatment following their booster revaccination on day 140. Gains in calves from all treatments were reduced from D 154 to D 168 due to a winter weather event with low temperatures and icy conditions. Calves in KM treatment were numerically (*p* = 0.19) lower than other treatments during this period. This reduced performance in KM was compensated for during the next period from D 168 to D 182, where KM tended (*p* = 0.09) to gain faster than MM and WN. Overall, postweaning ADG was not affected (*p* = 0.97) by vaccination timing or type of vaccine administered preweaning.

### 3.2. Bovine Respiratory Syncytial Virus SN Antibody Titers

**Figure 1:**
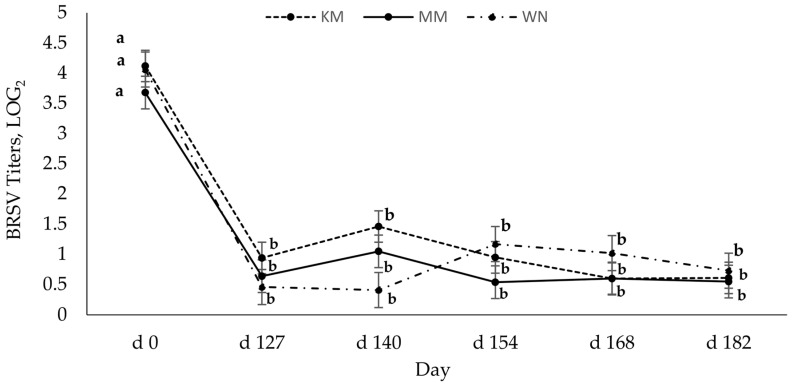
Least square means log_2_ BRSV antibody titers by day for calves vaccinated with either an inactivated BRD vaccine on D 0 (2 to 3 months of age) and a modified live viral vaccine (MLV) on D 127 or an MLV administered at d 0 and d 127 (weaning), or an MLV vaccine on d 127 (weaning) and d 140 (13 days post-wean). D 0 represents colostral antibodies. a, b Least square means with differing superscripts differ (*p* < 0.05). There was no effect of vaccination treatment or treatment × day interaction (*p* > 0.08), but the day effect was significant (*p* < 0.01).

The log-transformed means for serum-neutralizing (SN) antibodies to BRSV across days are presented in Figure 1. Results for neutralizing antibody titers against BRSV in calves on D 0 suggest that considerable colostral antibodies were present on D 0 at initial vaccination for KM and MM, and vaccine protection during the preweaning phase may have been minimal. Calves in both treatments were seronegative (SN < 2) by D 127, which was similar to the result reported on the WN treatment, which was unvaccinated until that time. No differences were observed at other time points (*p* > 0.14).

### 3.3. Bovine Viral Diarrhea Virus SN Antibody Titers

**Figure 2:**
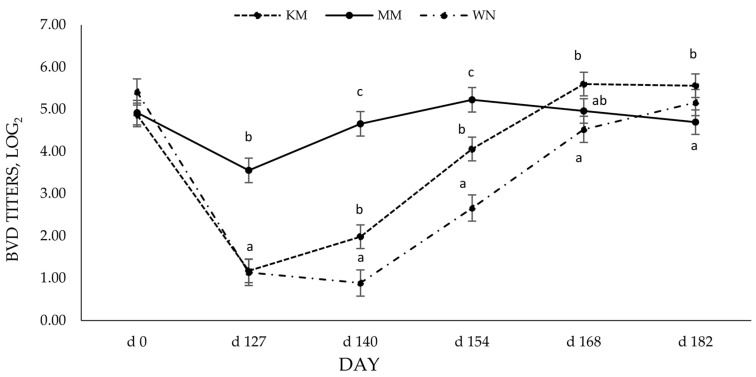
Least square means Log2 BVD antibody titers for treatment × day interaction in calves vaccinated with either an inactivated or modified-live viral BRD vaccine. Vaccines were administered for KM at d 0 (KV) and 127 (MLV), MM at d 0 (MLV) and 127 (MLV), and WN on d 127 (MLV) and 140 (MLV). KV = killed vaccine (ViraShield 6, Elanco Animal Health, Greenfield, IN). MLV = modified-live viral vaccine (Titanium 5, Elanco Animal Health, Greenfield, IN). a–c Least square means with differing superscripts differ by *p* < 0.05.

The SN antibody response to vaccination is presented in Figure 2. Serum-neutralizing antibody titers against BVDV-1 at d 0 (prior to initial vaccination) indicate passive transfer from dams; thus, no treatment differences were detected. However, on D 127, SN antibody titers in MM treatment were greater (3.48 ± 0.29, *p* = <0.0001) than in KM (1.16 ± 0.28) and WN (1.11 ± 0.31) groups. Additionally, at D 140 (13 days post-booster), SN antibody titers were greatest (*p* < 0.01) in the MM group. A strong anamnestic response was present in the KM group following revaccination with a MLV vaccine on D 127 and subsequently had the greatest (5.6 ± 0.28, *p* = 0.02) titer concentration by D 168, differing from the MM treatment. By the end of the 56-d preconditioning period, the mean across all treatment groups displayed sufficient SN antibody titers (>4, log2) to suggest protection against severe BVDV infection. 

### 3.4. ImmunoglobulinG and Subsets Response to Type and Timing of Vaccines

#### 3.4.1. Overall Effects of Treatment

**Figure 3:**
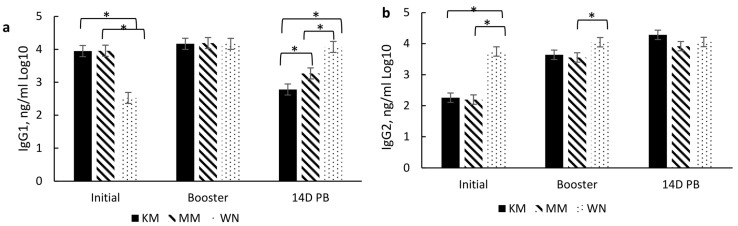
Effect of vaccination treatment by day of vaccination on serum concentrations (ng/ml) of immunoglobulin G1 (**a**) and immunoglobulin G2 (**b**) at initial vaccination, booster vaccination, and 14 days post booster vaccination (14D PB). For the KM and MM treatment groups, D 0 (initial), D 127 (booster), and D 140 (14D PB) correspond to day vaccination was administered, whereas, for the WN treatment group, D 127 (initial), D 140 (booster), and D 154 (14D PB). Data are expressed as means ± standard error of the mean. Means with * between treatments within a day differ at *p* < 0.05. Treatment × Day = *p* < 0.0001 for IgG1, *p* < 0.0001 for IgG2.

In this section, days refer to the time between initial vaccination, booster, and 14 days post booster (14D PB). A significant treatment × day of vaccination occurred for IgG1 (*p* < 0.0001) and IgG2 (*p* < 0.0001) concentrations (Figure 3) but not for total IgG or IgG1:IgG2 ratio (data not shown). The KM and MM groups had significantly higher IgG1 concentrations at initial vaccination than the WN group (Figure 3a). At 14D PB, IgG1 was 17% higher in MM than KM treatment group and 25% higher in WN than MM treatment group. Conversely, IgG2 concentrations were 66 and 70% higher at the initial vaccination time point in the WN group than KM and MM treatment groups, respectively (Figure 3b). However, by 14D PB, the KM group tended to have higher IgG2 than MM, while no difference was found between the KM and WN treatments.

Interestingly, IgG1 concentration increased by 5% from initial vaccination to booster and decreased by 33% from booster to 14D PB in the KM group. In contrast, in the MM treatment group, IgG1 concentration increased by 5% from initial vaccination to booster but decreased by 22% from booster to 14D PB. In the WN treatment group, IgG1 increased 65% between initial vaccination and booster and a 61% increase between initial vaccination and 14D PB. A similar pattern occurred for IgG2, with a 61% increase from initial vaccination to the booster and 89% from initial to 14D PB among the KM group. Additionally, IgG2 increased by 17% from booster to 14D PB among the KM group.

Similarly, IgG2 increased by 61% between the initial vaccination and booster and 78% between the initial vaccination and 14D PB in the MM treatment group. In contrast, there was no difference in IgG2 concentration in the WN group between days of vaccination. Overall, a main treatment effect occurred for total IgG (*p* = 0.042), with the WN group exhibiting significantly higher concentrations than the KM and MM groups (data not shown).

#### 3.4.2. Type of Vaccination and Timing of Vaccination

**Figure 4:**
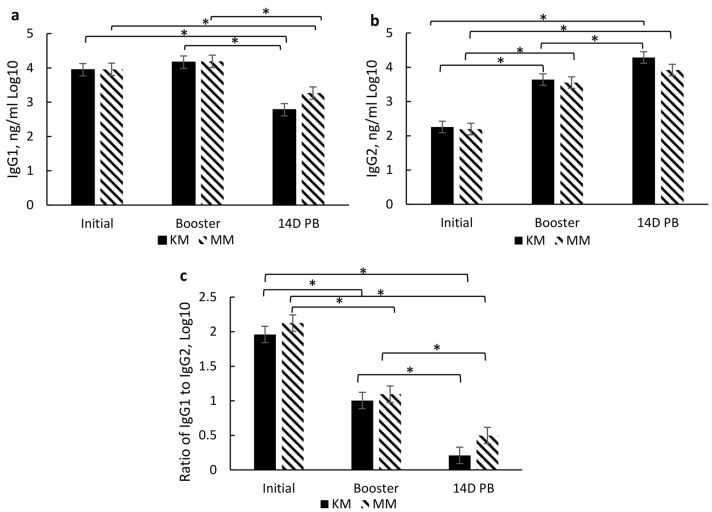
Effect of day of vaccination on serum concentrations (ng/mL) of immunoglobulin G1 (**a**), immunoglobulin G2 (**b**) and the ratio of immunoglobulin 1: immunoglobulin 2 (**c**) at initial vaccination, booster vaccination, and 14 days post booster vaccination (14D PB) for vaccination type. Data are expressed as means ± standard error of the mean. Means with * between days differ at *p* < 0.05. Day = *p* < 0.0001 for IgG1, *p* < 0.0001 for IgG2, and *p* < 0.0001 for IgG1:IgG2.

Due to vaccination protocol differences, further delineating differences between treatment groups may be more relevant to the effects of the type and timing of vaccination protocols. When comparing KM and MM treatments (similar time course, but different vaccine types initially administered), there was no significant treatment × day or treatment effects for IgG subsets. However, there was a day effect for IgG1 (*p* < 0.0001), IgG2 (*p* < 0.0001), and IgG1:IgG2 ratio (*p* < 0.0001), but not total IgG (*p* > 0.10). Regardless of treatment, IgG1 significantly decreased from initial vaccination to 14D PB (Figure 4a), while IgG2 significantly increased (Figure 4b), resulting in a decreased IgG1:IgG2 ratio (Figure 4c).

**Figure 5:**
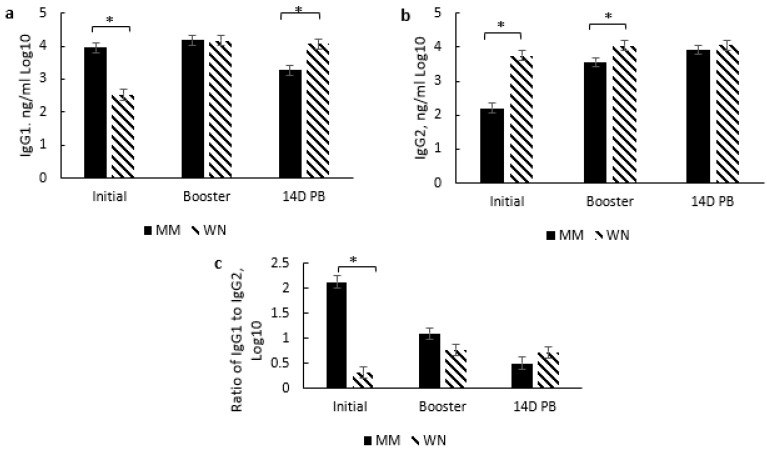
Effect of vaccination timing by day of vaccination on serum concentrations (ng/mL) of immunoglobulin G1 (**a**), immunoglobulin G2 (**b**), and the ratio of immunoglobulin 1 to immunoglobulin 2 (**c**) at initial vaccination, booster vaccination, and 14 days post booster vaccination (14D PB). Data are expressed as means ± standard error of the mean. Means with * between treatments within a day differ at *p* < 0.05. Treatment × Day = *p* < 0.0001 for IgG1, *p* < 0.0001 for IgG2 and *p* < 0.0001 for IgG1:IgG2.

When comparing the effect of administrating the same vaccination type, in this case, MLV, but on a different time course, a treatment × day of vaccination interaction occurred for IgG1 (*p* < 0.0001), IgG2 (*p* < 0.0001), and IgG1:IgG2 (*p* < 0.0001), but no effect on total IgG (*p* > 0.10). Specifically, IgG1 was 44% higher at the time of initial vaccination but 22% lower at 14D PB in the MM treatment group compared to the WN group (Figure 5a). Conversely, among the WN group, IgG1 concentration increased by 65% from initial vaccination to booster and 61% from initial vaccination to 14D PB. Moreover, IgG2 concentrations were 70% higher at initial vaccination and 13% at booster in the WN treatment group than in the MM treatment group (Figure 5b). At initial vaccination, the IgG1:IgG2 ratio was significantly higher in the MM group than in the WN treatment (Figure 5c). At the same time, no other significant differences occurred between treatment groups on the days of vaccination.

However, within treatment, IgG1 decreased by 32% between initial vaccination and 14D PB and by 61% between the booster and 14D PB in the MM group (Figure 5a). While IgG2 concentrations increased more than 420% between initial vaccination, booster, and 14D PB, in the MM treatment group (Figure 5b). The MM group also exhibited a significantly higher IgG1:IgG2 ratio at initial vaccination than at booster or 14D PB and booster than 14D PB, indicating an overall decrease in IgG1:IgG2 ratio (Figure 5c). Conversely, among the WN group, IgG1 concentration increased by 220% from initial vaccination to booster and 208% from initial vaccination to 14D PB (Figure 5a). The WN group had a significantly lower IgG1:IgG2 at initial vaccination than for samples obtained at booster and 14D PB (Figure 5c).

## 4. Discussion

Beef calves vaccinated for bovine respiratory disease (BRD) utilizing vaccination protocols that differed either in the type of vaccine (KM vs. MM) or timing of vaccination (MM vs. WN) displayed differential immune responses. Measures analyzed to characterize the immune response regarding vaccination type and timing included serum-neutralizing antibody titers to BRSV and BVD and serum concentrations of IgG1 and IgG2. Serum antibody titers have long been used to measure immunity from vaccination or natural infection [16,17] in the host animal. However, antibody titers as a parameter may be misrepresentative of disease protection. Vaccine-induced serum antibodies are a measurable response to immunogens [18,19] and vaccine efficacy. Passively acquired antibody titers of ≥256 have been shown to provide adequate protection against the manifestation of BVDV disease but not eliminate viral transmission [19]. Studies have analyzed the relationship between antibody titer concentration and protective immunity, but the results contradict. Animals with low to moderate antibody titers had greater protection post-challenges [17]. Conversely, others found low neutralizing titers associated with more severe clinical disease [19]. However, in our study, no challenge was applied to determine the level of protection due to vaccine treatment.

Regardless of vaccination type, host serum-neutralizing antibody titers for BRSV and BVD were highest preweaning (2 to 3 months of age), indicating the presence of maternal antibodies via passive immunity. Serum-neutralizing antibodies to BRSV significantly decreased from preweaning (day 0) and remained seronegative [20] for all animals throughout the study. It is plausible that the low BRSV titers may be due to maternal interference. Chase et al. [8] found that maternal antibodies can be present in the calf up to 6 months of age. Since maternal antibodies against viruses, especially BRSV, can interfere with the production of antibodies, thus reducing the antibody response [18,20], and it may take 40 days for maternal BRSV antibodies to decay [7], maternal interference likely contributed to low BRSV titers in these calves. Grooms and Coe [12] also observed that two doses of MLV, 21 days apart, generated the strongest antibody response, but BRSV titers waned rapidly. The reasons for this quick decline in BRSV titers post-vaccination or booster are not currently understood.

Although there was no effect of vaccine type on BRSV titers, serum-neutralizing antibody titers to BVD were differently affected by vaccine type. The calves receiving the modified live vaccine preweaning and at weaning (MM) had higher BVD titers following initial vaccination and 14 days post-booster. The longevity and level of antibody production that the initial dose of MLV vaccine provided was similar to previous reports by others that found MLV vaccines elicited a more vigorous response and greater duration of viable antibodies [16,18,20]. The more robust antibody response evoked by the MLV vaccine may explain the differences in BVD titers due to vaccine type. Modified live vaccines can induce a more effective titer response when maternal antibodies are present than killed vaccines [8], partly due to their ability to replicate in the host. The BVD titer response induced by the modified live vaccine (MLV) could imply that viral replication is important to induce a more robust antibody response, especially for BVD. Moreover, this may cause the higher BVD titers in the KM group at the end of the study. Others have shown that vaccination protocols containing at least one MLV result in higher serum-neutralizing antibodies to viruses within the BRD complex [21,22]. Still, others have shown a lack of antibody response to a single dose of a killed vaccine [12]. It should be noted that >90 days elapsed before the KM group received a booster, which goes beyond the manufacturer’s suggested vaccination protocol and may have contributed to differences in antibody titers.

In addition to serum-neutralizing antibody titers for BRD and BRSV, IgG subsets (IgG1 and IgG2) were analyzed to aid in characterizing the immune response to vaccination type and timing. These subsets are associated with a T helper 1 (Th-1, cell-mediated; IgG2) and T helper 2 (Th-2, antibody-mediated; IgG1) immune response, respectively [23] as cytokines associated with either a Th-1 or Th-2 response aid in B lymphocyte signaling for the increase of these subsets. Moreover, the IgG1:IgG2 ratio indicates a bias toward a Th-1, a Th-2, or a balanced response. A ratio between 0.5 and 2 indicates a balance between Th-1 and Th-2, while <0.5 indicates a Th-1 bias, and >2 indicates a Th-2 [24]. Thus, induction of both immune responses may be important for the clearance and protection of BRD [25,26]. However, vaccine type did not affect IgG subsets or the IgG1:IgG2 ratio, but there was a day effect which may also be due to passive immunity. Higher IgG1 concentrations prior to initial vaccination in the KM and MM treatment groups may be due to maternal influence, especially since IgG1 is the predominant subset that passes through the dam’s milk [27] and the fact that the WN treatment group had reduced IgG1 by the time of their initial vaccination at weaning. The IgG1 concentration did wane over the sampling period, which indicates that maternal influence wanes, which most likely occurs by weaning. The decrease in IgG1 and increase in IgG2 resulted in a reduced IgG1:IgG2 ratio from the day of initial vaccination (2 to 3 months of age) through 14 days post-booster (d 140). The combination of these changes and an IgG1:IgG2 ratio of <0.05 indicates a shift from an antibody-mediated response to a cell-mediated response over the vaccination time course. It is plausible that this is partly due to the duration of time between initial vaccination and revaccination.

Interestingly, the IgG subset and ratio were differentially affected by the timing of the initial and booster among the groups that received two doses of the modified live vaccine. The MM group exhibited higher IgG1 levels at initial vaccination, while the WN group had higher IgG2 levels at initial vaccination and booster sample days. These results indicate a possible bias of the MM group towards a Th-2 (antibody-mediated) response at the initial vaccination, though the response appears more balanced at subsequent sample time points. In contrast, the WN group may be more skewed towards a Th-1 (cell-mediated) response at the initial vaccination while also appearing to move towards a more balanced response at subsequent sample time points. These differences due to the timing of vaccine administration may also be partly due to maternal influence among the MM group, as IgG1 is prevalent in milk [27]. Conversely, the WN treatment group appears biased towards a Th-2 response, though both treatment groups’ IgG1:IgG2 ratios indicate a Th-1/Th-2 balance by the booster vaccination [24]. This skew from antibody-mediated immunity may partly explain a delay in specific antibody production for BVD.

At weaning (D 127), the MM group still exhibited significantly higher BVD-neutralizing antibodies than the WN group. Moreover, despite increased BVD antibodies for the WN group, the MM remained elevated from 14 days post booster (D 140) to 28 days post booster (D 154). WN group antibodies did not reach higher levels than MM until 28 days post their booster vaccination (D 182). Grooms and Coe [12] also found that calves receiving an MLV at pre-wean and weaning had higher BVDV titers following the booster than calves that received an MLV at weaning and then again 3 weeks later. The delay in antibody production to BVD from initial to booster vaccination should also be noted. It is plausible that the immune response was dampened by the occurrence of weaning for the WN group. Weaning acts as a stressor both physically and psychologically, and chronic stress can have immunosuppressive effects [3]. These results indicate that the timing of vaccination administration, especially in the face of maternal interference and weaning, may influence the immune response. This effect seems more significant in response to initial vaccination than subsequent vaccinations.

Despite these differences in BRD titers, IgG1 and IgG2, there were no effects of treatment on body weight, and there was minimal effect on ADG. Among the WN treatment group, ADG slightly decreased at 14 days post-booster, and KM calves had slightly higher ADG at the end of the study. Richeson [10] found that highly stressed steers vaccinated upon receiving gained less early in receiving than steers whose BRD vaccination was delayed. Others have reported no effect on body weight or ADG in calves vaccinated for BRD during the preconditioning or receiving periods [9,28,29,30]. Though slight differences were found between treatment types and vaccination timing, the effect on vaccination efficacy and protection remains unknown. Additional sampling time points, as well as viral challenges, would aid in elucidating if multiple factors, such as the presence of maternal interference, animal age, vaccine type, and time between vaccination and booster, affect the ability of the vaccine to reduce disease occurrence and severity.

## 5. Conclusions

Treatment did not affect BW but transiently differed in ADG. However, some differences in ADG may have been due to responses to weather challenges. Administration of at least one MLV vaccine appeared to induce a more robust antibody response, though utilizing the MLV as the initial vaccination may help overcome maternal interference. While vaccination with an MLV at weaning did produce a robust antibody response, it may be delayed by stress and thus have reduced efficacy. Further field-based research investigating antibody production to vaccine type and vaccination timing concerning the level of protection afforded and the future immune status and performance through subsequent beef industry segments are needed to develop the most effective respiratory vaccination protocols.

Ultimately, vaccination timing depends on the goals of the operation. This information is of the utmost importance given a recent review and meta-analysis [31] that suggested that vaccines used at or near feedlot arrival did not reduce the incidence of BRD. Research that ties the two industry segments is limited, examining preweaning vaccination strategies and subsequent performance in feedlots. Such research would require a large amount of time and resources for a large sample size to produce significant results. However, research that links the two segments of the industry is necessary.

## Figures and Tables

**Table 1:** Effect of vaccination type and timing of initial vaccination on growth performance of beef calves.

	Treatment				*p* Value		
Item	KM ^1^	MM ^2^	WN ^3^	SE	Sex	Trt	Sex × Trt
Body weight, kg							
Day 0	103	103	103	2.8	0.03	0.99	0.82
Day 127	244	244	244	8.4	<0.01	0.99	0.39
Day 140	250	251	252	9.2	<0.01	0.94	0.68
Day 154	259	263	257	10.3	<0.01	0.70	0.61
Day 168	262	267	264	10.0	<0.01	0.74	0.74
Day 182	276	276	275	7.4	<0.01	0.99	0.56
Average Daily Gain, kg							
Day 0 to 127	1.11	1.11	1.11	0.036	<0.01	0.97	0.20
Day 127 to 140	0.48	0.58	0.62	0.356	0.94	0.63	0.16
Day 140 to 154	0.73	0.81	0.43	0.144	0.52	0.08	0.19
Day 154 to 168	0.19	0.33	0.48	0.133	0.50	0.19	0.13
Day 168 to 182	0.96	0.60	0.78	0.233	0.06	0.09	0.39
Day 127 to 168	0.59	0.58	0.58	0.057	0.81	0.97	0.88

^1^ KM = killed vaccine (KV) administered preweaning on D 0 (2 to 4 months of age) with modified-live viral vaccine (MLV) booster on D 127 (weaning).^2^ MM = MLV vaccine administered preweaning on D 0 (2 to 4 months of age) and a booster on D 127 (weaning).^3^ WN = MLV vaccine administered on D 127 (weaning) and D 140 (13 days post-weaning).

## Data Availability

Data presented in this study are available on request from the corresponding authors.
